# Differentiated Contact Tracing for Emerging Zoonotic Hantavirus and Ebolavirus Infections: Lessons from the 2026 Andes Virus (MV Hondius) and Bundibugyo Virus Outbreaks

**DOI:** 10.3390/v18070708

**Published:** 2026-06-26

**Authors:** Francesco Broccolo, Massimiliano Galdiero

**Affiliations:** 1 UOSD Virology and Microbiology, University Hospital P.O. Vito Fazzi—ASL Lecce, 73100 Lecce, Italy; 2 Department of Experimental Medicine (DiMeS), University of Salento, 73100 Lecce, Italy; 3 Department of Woman, Child and General and Specialized Surgery, University of Campania Luigi Vanvitelli, 80138 Naples, Italy; massimiliano.galdiero@unicampania.it; 4 UOC Virology and Microbiology, University Hospital “Luigi Vanvitelli”, 80138 Naples, Italy

**Keywords:** Andes virus, MV Hondius, Bundibugyo virus, contact tracing, active surveillance, presymptomatic transmission, asymptomatic transmission, person-to- person transmission, RT-qPCR diagnostics, favipiravir, tocilizumab, hantavirus vaccine

## Abstract

In May 2026, two outbreaks occurred at once: an Andes virus outbreak aboard the MV Hondius expedition cruise ship (13 cases (12 laboratory-confirmed and 1 probable), 3 deaths, and more than 600 contacts traced across 32 countries and territories; passengers and crew from 23 countries; data as of 22 June 2026) and a Bundibugyo virus disease (BDBV) outbreak in Central Africa (more than 1000 laboratory-confirmed cases and more than 250 confirmed deaths in the Democratic Republic of the Congo and Uganda; data as of 22 June 2026). The two pathogens diverge sharply in their transmission epidemiology. Aboard the MV Hondius, person-to-person transmission was sustained across three generations from a single index case, and one asymptomatic, PCR-positive contact later developed symptoms, indicating pre-symptomatic ANDV RNA detection, although the extent to which this corresponds to infectiousness and onward transmission remains uncertain. BDBV, on current evidence, has not been documented to sustain pre-symptomatic transmission as a major driver of its outbreaks, which allows for contact tracing of different timing and intensity. This Perspective draws together the epidemiological data from the MV Hondius outbreak, the endemic-region experience (Chile’s 2021–2025 secondary attack rate of 5.7%; Argentina’s 1996 El Bolsón and 2018 Epuyén outbreaks), the molecular pathogenesis, the diagnostic platforms, the therapeutic pipeline (favipiravir, tocilizumab, Icatibant) and the vaccine strategies (USAMRIID DNA vaccine, phase 1 completed). We set the contact-tracing and active-surveillance protocols applied to ANDV against the less stringent protocols that may suffice for BDBV.

## 1. Introduction

The global landscape of published hantavirus research comprises 4561 peer-reviewed articles indexed on PubMed, with 178 publications focused specifically on Bundibugyo virus—a disproportion reflecting the concentration of funding and attention on pathogens seen as established threats rather than on emerging zoonotic viruses with pandemic potential [1]. The two outbreaks of May 2026—the MV Hondius Andes virus (ANDV) event and the Central African Bundibugyo virus disease (BDBV) epidemic—run against that imbalance and call for a careful, evidence-based appraisal of both pathogens [2,3].

The MV Hondius outbreak is the largest documented cluster of Andes virus cases outside South America: 13 cases (12 laboratory-confirmed and one probable), 3 deaths and more than 600 contacts traced across 32 countries and territories, originating on an expedition cruise ship carrying passengers from 23 countries (data as of 22 June 2026) [2]. Transmission ran person-to-person across three generations from a single index case, produced two distinct epidemic peaks over an 18-day period, and included an asymptomatic, PCR-positive contact who later developed symptoms [2]. This presymptomatic viremia bears directly on how contact tracing should be timed and resourced.

In the same weeks, the WHO declared a Public Health Emergency of International Concern (PHEIC) for BVD in the Democratic Republic of the Congo and Uganda, recording more than 1000 laboratory-confirmed cases and more than 250 confirmed deaths across the two countries as of 22 June 2026 (Democratic Republic of the Congo: 1003 confirmed cases and 254 confirmed deaths; Uganda: 20 confirmed cases and 2 deaths; case-fatality ratio among confirmed cases approximately 24%), with transmission concentrated in the Ituri, North Kivu and South Kivu provinces [3]. The available data suggest that BDBV has not been documented to sustain pre-symptomatic transmission as a major driver of its outbreaks in the way reported for ANDV, which permits a different allocation of resources. Unless otherwise indicated, all epidemiological figures in this Perspective Study are reported as of a data cut-off of 22 June 2026 and are drawn from WHO, ECDC, Africa CDC and national public-health authority reports; case classifications (confirmed, probable, suspected) follow the definitions used by those sources.

These two pathogens are not compared here because they are biologically alike—they are not. ANDV is an orthohantavirus and BDBV an orthoebolavirus, and they differ fundamentally in reservoir, tissue tropism, transmission ecology, biosafety requirements and the response infrastructure they demand. They are compared because they occupy contrasting positions on a single operational axis that governs outbreak response: the timing of infectiousness relative to symptom onset, the feasibility of pathogen-specific diagnostics, and the outbreak-response capacity of the affected setting. Read along this axis, the very differences between the two viruses become the reason their contact-tracing intensity, diagnostic strategy and resource allocation must differ—a framework intended to be transferable to future emergences rather than specific to the events of 2026.

## 2. Contact Tracing and Active Surveillance: Critical Divergence Between ANDV and BDBV

### 2.1. ANDV: Pre-Symptomatic RNA Detection and the Case for Intensive Active Surveillance

The MV Hondius outbreak made it possible to document ANDV transmission dynamics in real time. One contact tested PCR-positive while still asymptomatic and only later developed symptoms, which shows that detectable viremia precedes symptom onset [1,2]. Whether this pre-symptomatic RNA detection corresponds to infectious virus and to onward transmission before symptom onset remains uncertain; nonetheless, it defines the window that contact tracing aims to cover.

The biological basis for this lies in a nucleoprotein function unique to ANDV that sustains replication in the respiratory epithelium and prolongs shedding into saliva and respiratory secretions (see Section 4.1); this property is not shared by other hantaviruses and may contribute to the capacity for sustained person-to-person spread observed for ANDV. Such transmission is, however, likely multifactorial, shaped by viral load, disease phase, the duration and proximity of contact, household and indoor exposure, host factors and outbreak setting, rather than attributable to any single molecular determinant [4,5].

A prospective study of shedding dynamics found transmission risk to be highest in the prodromal and early symptomatic phases, when infectious virus is recoverable from oral and respiratory secretions [6]. Viraemia (RNA) is detectable 1–3 days before symptoms begin; whether this pre-symptomatic RNA detection corresponds to infectious virus and to onward transmission before symptom onset has not been established, so the possibility is treated here as a precautionary concern rather than a demonstrated route [2,6].

These dynamics support a precautionary, risk-based and resource-dependent approach to contact tracing for ANDV. WHO interim guidance of 15 May 2026, together with endemic-region experience from Chile, sets out what this requires: daily symptom monitoring of all close contacts; serial RT-PCR at baseline, day 3 and day 7, with further testing if symptoms appear; and a monitoring or quarantine period of up to 42 days (six weeks) after the last exposure, consistent with the long and variable ANDV incubation period [2,6,7]. Chile’s 2021–2025 programme followed 106 close contacts with daily checks and serial RT-PCR and found 6 secondary cases (a secondary attack rate of 5.7%), all within households [2]. Consistent with this resource-dependent logic, these measures are intended to be applied in a risk-stratified manner. In the MV Hondius response, contacts were classified as higher-risk or lower-risk (approximately 53% and 47% of those traced, respectively): higher-risk contacts—household members, those with prolonged close or indoor exposure, and caregivers—are the priority group for quarantine and serial RT-PCR, whereas lower-risk contacts may reasonably be managed with daily symptom monitoring alone and tested only if symptoms develop. This stratification, rather than universal serial testing of all asymptomatic contacts, is what the precautionary approach entails.

### 2.2. BDBV: Pre-Symptomatic Transmission Not Documented as a Major Driver, Within a Broader Ebola Response Framework

Bundibugyo virus disease looks epidemiologically different. Pre-symptomatic or asymptomatic transmission has not been documented as a major driver of BDBV outbreaks, in contrast to the dynamics reported for ANDV [3,8,9]. Importantly, this does not reduce BDBV contact tracing to symptomatic individuals: it must be conducted within the full Ebola-disease response framework, encompassing healthcare workers and family caregivers, funeral and direct body-fluid exposure, cross-border population movement, community engagement, and the security constraints that shape field operations. The difference from ANDV is therefore one of the relative timing and intensity of laboratory testing, not of the overall scope of the response.

The 2007 Uganda BDBV outbreak involved 149 suspected cases and 37 confirmed deaths (case fatality rate ~25%); secondary transmission there came mainly from symptomatic, hospitalized patients in the acute phase, not from presymptomatic or asymptomatic contacts, and the few secondary cases were confined to healthcare workers and family members exposed to acutely ill patients [10].

WHO guidance of May 2026 therefore allows for a more resource-conservative approach for BDBV: symptom monitoring from the last exposure through day 21; RT-PCR reserved for symptomatic contacts, with baseline testing of asymptomatic contacts left optional; and a 21-day quarantine after the last exposure—a longer window, given the variable incubation period—without the intensive daily surveillance or serial PCR in asymptomatic individuals that ANDV demands [3,9]. This matters in Central African settings with limited laboratory capacity and ongoing insecurity [3] (Table 1).

### 2.3. Geographic and Healthcare Setting Implications

The contrast between the two contact-tracing strategies plays out differently depending on the setting.

In settings with laboratory capacity, such as the MV Hondius, ANDV can be contained through intensive presymptomatic surveillance. The ship reached rapid diagnosis and isolation of its 13 cases and allowed the transmission chains to be investigated, though the presymptomatic window still requires a 24–48 h turnaround on RT-PCR results, which is hard to guarantee in rural regions [2].

BDBV, by contrast, spreads in settings marked by limited laboratory capacity, insecurity and a strained health system, as in the 2007 Uganda outbreak and the current DRC epidemic. Because presymptomatic transmission appears absent, contact tracing can concentrate on symptomatic individuals and on healthcare workers with direct exposure to body fluids, instead of screening every contact by presymptomatic RT-PCR [3,9].

## 3. Epidemiology: MV Hondius Outbreak and Endemic-Region Context

### 3.1. The MV Hondius Outbreak: Person-to-Person Transmission and Epidemic Dynamics

Among ANDV clusters reported outside South America, the May 2026 MV Hondius event stands out as the most consequential from an epidemiological standpoint [1,2]. Aboard the expedition cruise ship, 13 cases were recorded (12 laboratory-confirmed and one probable) and 3 proved fatal, giving a case-fatality rate of 23% (3/13 including the probable case, or 25% if restricted to the 12 confirmed cases); public health teams traced more than 600 contacts across 32 countries and territories among passengers and crew from 23 countries, as of the 22 June 2026 data cut-off [1,2]. What made the cluster exceptional was its chain structure: a single index case seeded transmission that propagated through as many as three successive human generations, producing two separate case peaks over an interval of roughly 18 days [2].

Infectiousness concentrated in the prodromal and early symptomatic window, the period in which replicating virus can be recovered from oral and respiratory secretions [2,6]. On 15 May 2026 a WHO-convened emergency scientific consultation brought experts together to evaluate the event and set research priorities, distilling three pressing conclusions: that ANDV is capable of self-sustaining human-to-human spread, that the medical countermeasure pipeline is still underdeveloped, and that the scientific networks already working on the virus need immediate reinforcement [1,2].

### 3.2. Endemic-Region Epidemiology: Chile and Argentina Experience

The Chilean experience with ANDV, accumulated over many years, provides valuable operational insight into both outbreak control and the way the virus moves between people. In the Los Ríos region, public health authorities followed 106 close contacts of confirmed cases across 2021–2025, combining daily symptom surveillance with serial RT-PCR [2]. The follow-up turned up six secondary infections, for a secondary attack rate (SAR) of 5.7% [2]. Every one of those six arose within a household, which points to domestic exposure as the main route of transmission [1,2].

Argentine genomic work has added further resolution to these transmission dynamics and to the question of viral evolution. Human-to-human spread was first demonstrated during the 1996 El Bolsón outbreak, which recorded 16 cases and 9 deaths—a case fatality rate of 56% [11]. The 2018 Epuyén outbreak (34 cases, 11 deaths; 32% case fatality rate) traced back to a birthday party, and 64% of all transmission events were linked to that single event [12]. Contact chains in this outbreak ran through four successive human generations, with the reproductive number (R0) estimated at about 2.1 until control measures brought spread down [12]. Sequencing revealed little genomic variation—the mutations detected were exclusively synonymous—implying that the spread was driven by human behaviour rather than by adaptive change in the virus [12] (Table 2).

## 4. Molecular Pathophysiology and Tissue Tropism

### 4.1. ANDV: Innate Immune Evasion and Pulmonary-Centric Pathogenesis

The pulmonary syndrome (HPS) caused by Andes virus arises from its tropism for endothelium, where infection raises vascular permeability selectively without destroying the endothelial cells themselves [13]. At the molecular level, what sets ANDV apart is a nucleoprotein (NP) function that blocks phosphorylation of interferon regulatory factor 3 (IRF-3); this blockade permits the virus to keep replicating in respiratory epithelium and to maintain prolonged shedding into saliva and respiratory secretions [4,5]. This property is not described for Sin Nombre virus or the Old World hantaviruses and may be one of several factors contributing to the comparatively greater transmissibility of ANDV, rather than its sole determinant [4].

The progression of ANDV-associated HPS can be summarized in four overlapping stages: endothelial infection in which non-lytic replication drives capillary leakage confined to the pulmonary bed; interstitial fluid accumulation; an immune phase that begins as anti-ANDV IgM and IgG appear (roughly days 5–14 after symptom onset); and thrombocytopenia accompanied by disordered platelet aggregation [14,15]. Clinically the trajectory is difficult to predict, frequently strikes otherwise healthy adults in their thirties and forties, and may collapse abruptly into respiratory failure severe enough to require extracorporeal membrane oxygenation (ECMO) [2].

Cytokine work has begun to resolve severe ANDV disease into separate inflammatory patterns. One line of evidence frames it as an interleukin-6 (IL-6)-driven syndrome—the rationale behind compassionate-use tocilizumab (an IL-6 receptor blocker) in critically ill patients [16]. The profiling of 33 ANDV patients pointed to two further mechanisms: an interferon-dominated endothelial hyperinflammation marked by elevated IP-10, CXCL9, and IL-18; and a bradykinin-mediated vascular-leak pathway tied to platelet and contact-system activation with raised D-dimer and potentially amenable to the bradykinin-receptor antagonist Icatibant [2].

### 4.2. BDBV: Glycoprotein-Mediated Molecular Divergence from Zaire

Bundibugyo virus represents a distinct ebolavirus species with substantial sequence divergence from Zaire ebolavirus. Complete genome analysis reveals 32% nucleotide divergence and 35% amino acid divergence specifically in the surface glycoprotein (GP) [17]. This glycoprotein divergence is functionally significant: the ebolavirus GP is the sole determinant of cell tropism and viral attachment. The 35% divergence implies non-conservative substitutions across critical binding domains, potentially altering receptor engagement efficiency and contributing to BDBV’s comparatively lower case fatality rate (30–50% historical) versus Zaire (50–90%) [17,18] (Table 3).

## 5. Diagnostic Platforms and WHO Guidance

### 5.1. ANDV Diagnostics: WHO Interim Guidance and Limitations

For ANDV, RT-PCR performed on whole blood is still the reference diagnostic approach [1,2]. The difficulty is one of availability: dedicated ANDV assays are scarce on the commercial market, since most marketed hantavirus tests were designed around Old World species [1,2]. The WHO addressed this gap in interim guidance released on 15 May 2026, advising that, in the absence of an ANDV-specific assay, laboratories fall back on generic hantavirus RT-PCR directed at conserved S and/or L genomic segments and then confirm the species by sequencing [19]. In practice, much of the outbreak response leaned on in-house protocols from established Chilean and Argentine laboratories, with WHO coordinating the rapid exchange of primers, controls and biosafety information [2].

Broadening access to validated commercial tests for New World hantaviruses is therefore an urgent need [2]. The principal shortcomings at present are threefold: many commercial kits do not disclose their primer and probe sequences; clinical validation against the European ANDV isolate is lacking; and inter-laboratory proficiency testing remains limited [20].

### 5.2. BDBV Molecular Diagnostics: L-Gene Assays Required

The central diagnostic challenge for BDBV is that Zaire-specific RT-qPCR assays fail to reliably detect BDBV due to the 35% glycoprotein sequence divergence [17]. Evidence for this diagnostic failure is documented in the 2007 BDBV discovery: samples were negative when tested with Zaire- and Sudan-specific RT-qPCR assays, but positive when tested with broadly reactive ebolavirus antigen-capture ELISA and subsequent pyrosequencing [10]. The specimens carried high-titre BDBV RNA, so the species-specific assays were returning false negatives [10].

L-gene targeted assays (such as Altona RealStar Filovirus Screen RT-PCR Kit 1.0, CE-IVD marked) successfully detect BDBV, as the L gene exhibits greater sequence conservation across ebolavirus species [21]. During the May 2026 outbreak, INRB Kinshasa confirmed viral RNA using the Altona assay in 8 of 13 clinical samples [3,22] (Table 4).

## 6. Therapeutic Pipeline: Current Development Status

### 6.1. ANDV Antivirals: Favipiravir and Ribavirin Status

No therapy is currently approved for ANDV, although work on medical countermeasures is actively progressing [2]. The repurposed antiviral furthest along is favipiravir, whose case rests on three elements: activity demonstrated in vitro, efficacy observed in animal models, and a human safety record accumulated during COVID-19 and other viral indications [2]. In vitro and animal-model activity, together with safety data from other indications, do not in themselves establish clinical efficacy against ANDV; its use therefore remains investigational, anticipated chiefly for post-exposure prophylaxis and the earliest phase of illness [2].

Ribavirin, by contrast, has been set aside, owing to uneven efficacy data and unresolved safety questions [2]. A mortality reduction in ANDV-HPS (reported from around 50% to roughly 38–40% when given early) has been described, but the supporting evidence is limited by study design and disease context, the effect is judged insufficient on current data, and other strategies are preferred [23]. Additional agents such as molnupiravir and baloxavir still await urgent in vitro testing, and monoclonal antibodies, though promising, have not advanced beyond the preclinical stage [2].

### 6.2. Immunomodulators: Tocilizumab and Icatibant

Once the disease has progressed to cardiopulmonary involvement, the therapeutic emphasis shifts away from antivirals toward immunomodulators aimed at the inflammatory response or at vascular leakage [2]. Recent immunological profiling points to two candidates.

The first is tocilizumab, an IL-6 receptor blocker. Severe ANDV disease appears to involve an IL-6-driven inflammatory syndrome, and the drug has been used on an investigational, compassionate-use basis in critically ill patients to blunt that hyperinflammation, without controlled efficacy data in ANDV [2,16].

The second is icatibant, a selective bradykinin B2 receptor antagonist. Cytokine profiling of 33 ANDV patients identified a bradykinin-mediated vascular-leak pathway—marked by raised D-dimer and by platelet and contact-system activation—that icatibant could, in principle, target; this remains an investigational hypothesis rather than an established treatment [2] (Table 5).

## 7. Vaccine Development: Phase 1 Data and Current Status

### 7.1. ANDV Vaccine: USAMRIID DNA Vaccine Phase 1 Completion

Vaccine efforts against ANDV remain predominantly at the discovery and early-development stage. The frontrunner is a multi-dose DNA vaccine from the United States Army Medical Research Institute of Infectious Diseases (USAMRIID), built around the viral glycoprotein [2]. Two results define its current status: antibody-mediated protection in hamster challenge models, and a completed phase 1 human study reporting safety and immunogenicity data [24]. Viral-vector and mRNA candidates exist as well but have not progressed past preclinical work [2].

Several questions still cloud the path forward, notably whether protection cross-neutralizes across ANDV lineages and how to handle the so-called ‘hamster paradox’—the observation that rodent-derived strains kill in animal models while human-derived strains do not, which complicates the design of efficacy studies [2].

### 7.2. BDBV Vaccine: Investigational Approaches

No vaccine exists for BDBV. Given the 35% glycoprotein divergence between BDBV and Zaire ebolavirus, existing Zaire-based vaccines (ERVEBO, Zabdeno/Mvabea) may afford cross-protection that is uncertain and likely limited; confirmation requires BDBV-specific immunogenicity and protection data [3]. Investigational BDBV vaccines employ platforms analogous to those in other ebolaviruses, namely, recombinant GP-based subunits, viral vector-based platforms, and viral-like particle (VLP) systems, though all of these are still in preclinical development, and the ongoing Central African epidemic provides both an ethical imperative and a clinical trial window for accelerated development [3] (Table 6).

## 8. Conclusions and Recommendations

The May 2026 MV Hondius and Central African BVD crises expose critical gaps in global preparedness for pathogens known for decades to cause severe disease and documented person-to-person transmission. The MV Hondius outbreak demonstrated sustained ANDV transmission across three generations with international spread, while the BVD epidemic unfolded in a region with limited diagnostic and therapeutic infrastructure.

The distinction this Perspective Study draws has been largely overlooked: because ANDV shows pre-symptomatic RNA detection, whose correspondence to infectiousness remains uncertain, a precautionary, risk-based approach favours more intensive contact tracing and serial RT-PCR of asymptomatic individuals; for BDBV, where pre-symptomatic transmission has not been documented as a major driver, a more resource-conservative approach that prioritizes symptomatic contacts while retaining the full Ebola response framework is reasonable. That difference shapes how outbreaks should be handled in different geographic and health-system settings.

Several priorities follow directly. For ANDV, endemic and outbreak-affected regions need presymptomatic surveillance protocols built around a 24 h RT-PCR turnaround, while for BDBV, contact tracing can reasonably be concentrated on symptomatic individuals where resources are scarce. Diagnostics need validating and broadening so that commercial assays cover ANDV specifically and detect BDBV reliably. On treatment, favipiravir trials for early ANDV disease should be accelerated and clear protocols should be established for the immunomodulators, tocilizumab and icatibant, used in severe disease. On prevention, the USAMRIID ANDV DNA vaccine should move into phase 1b/2 trials, and partnerships should be set up to develop a BDBV vaccine. Underpinning all of this is sustained investment in the research networks and frontline teams in endemic countries that already have significant experience of both viruses.

A collaborative structure is already in place: the International Society of Hantaviruses, founded in 1989 and now chaired by Nicole Tischler, could be reinforced to speed progress on diagnostics, therapeutics and vaccines [2]. Future preparedness depends on translating this momentum into sustained investment rather than reactive crisis response.

## Figures and Tables

**Table 1:** Contact-tracing and active surveillance protocols for ANDV vs. BDBV.

Parameter	ANDV (MV Hondius Model)	BDBV (Central African Model)
Presymptomatic transmission	Pre-symptomatic RNA detection DOCUMENTED (RNA detectable 1–3 days before symptom onset; contact PCR+ while asymptomatic); transmission before symptom onset NOT ESTABLISHED [2]	NOT DOCUMENTED as a major driver: limited pre-symptomatic shedding reported; transmission mainly from symptomatic patients [10]
Symptom monitoring	Daily checks from day 0 through day 42 (6 weeks) post-last exposure [2,7]	Daily checks recommended; can reduce to periodic if asymptomatic after day 5 [3,9]
RT-PCR in asymptomatic contacts	Baseline + serial (day 3, day 7) recommended on a risk-based, precautionary basis given pre-symptomatic RNA detection [2,6]	Optional baseline; prioritize RT-PCR for symptomatic contacts [3]
Quarantine duration	Up to 42 days (6 weeks) post-last exposure (long incubation period) [2,7]	21 days post-last exposure (longer incubation window) [3,9]
Secondary Attack Rate (SAR)	5.7% (all household contacts; Chile 2021–2025) [2]	Variable; 2007 outbreak showed transmission primarily from symptomatic patients [10]
Resource requirements	HIGH: Requires laboratory capacity for serial RT-PCR in asymptomatic individuals	MODERATE: comprehensive contact tracing and monitoring of all defined contacts; laboratory testing (RT-PCR) prioritized for contacts who develop compatible symptoms
Setting	Developed countries with laboratory capacity (e.g., MV Hondius)	Resource-limited Central Africa with limited lab infrastructure

**Table 2:** Comparative epidemiology of ANDV vs. BDBV.

Parameter	ANDV	BDBV
Current Outbreak	MV Hondius (as of 22 Jun 2026): 13 cases (12 confirmed, 1 probable), 3 deaths, 600+ contacts across 32 countries/territories, 3 generations transmission [2]	Central Africa (as of 22 Jun 2026): >1000 confirmed cases, >250 confirmed deaths (CFR ~24% among confirmed); DRC 1003 confirmed/254 deaths, Uganda 20 confirmed/2 deaths [3]
Case Fatality Rate (CFR)	MV Hondius: 23% (3/13 total cases; 25% if restricted to 12 confirmed); Endemic: 35–40% [2]; El Bolsón 1996: 56% (9/16); Epuyén 2018: 32% (11/34) [11,12]	2007 Uganda: 25%; 2012 DRC: 50%; 2026 DRC/Uganda: 25.4% suspected, 12.1% confirmed [3,10]
Secondary Attack Rate (SAR)	Chile 2021–2025: 5.7% (all household contacts) [2]	Not clearly defined for recent outbreak; 2007 mainly healthcare/family contacts [10]
Reproductive Number (R0)	Argentina 2018 Epuyén: ~2.1 before control measures [12]	Not estimated; limited sustained chains observed [10]
Transmission Setting	Household contacts (94% of SAR in Chile) [2]; airborne prodromal/early symptomatic [6]	Healthcare workers & direct fluid contact during acute illness [10]
Generations Documented	Up to 4 (Argentina 2018); up to 3 in cruise ship [12]; asymptomatic case documented [2]	Limited secondary chains; no presymptomatic transmission documented [10]

**Table 3:** Molecular pathophysiology: ANDV vs. BDBV vs. other hantaviruses.

Feature	ANDV	Sin Nombre Virus (SNV)	Old World (PUUV/HTNV)
Syndrome	HPS (pulmonary)	HPS (pulmonary)	HFRS (renal)
NP IRF-3 suppression	YES (unique)—enables persistent replication [4,5]	NO—acute clearance [4]	NO—acute clearance [4]
Tissue tropism	Pulmonary endothelium (primary)	Pulmonary endothelium	Renal tubules (primary)
Salivary/respiratory shedding	Sustained >14 days—documented [2,6]	Limited—no human-to-human [4]	Not documented [4]
Human-to-person transmission	DOCUMENTED sustained [2,12]	NO—never documented [4]	NO—never documented [4]
Case fatality rate	35–40% endemic; MV Hondius 23% (3/13 total cases; 25% if 3/12 confirmed) [2]	~38–40% [4]	PUUV < 1%; HTNV 5–15% [4]

**Table 4:** Diagnostic assays for ANDV and BDBV.

Assay/Target	Performance and Notes (Data Cut-Off 22 June 2026)
**ANDV (Hantaviridae)—separate diagnostic pathway**	
Whole-blood RT-PCR (reference method)	Reference method; highest yield from the buffy-coat fraction [2,20]
ANDV-specific RT-qPCR	Recommended; limited commercial availability; in-house protocols from Chile/Argentina [2,19]
Hantavirus-generic RT-PCR (conserved S/L segments)	Use per WHO interim guidance when ANDV-specific assays are unavailable; confirm species by sequencing [19]
S/L-segment sequencing	Species confirmation [19]
Serology (IgM/IgG)	Supports diagnosis; yield depends on timing relative to symptom onset [2]
Specimen-type limitation	Whole blood/buffy coat preferred; viraemia may be short-lived in mild cases [2,6]
**BDBV (ebolavirus, Filoviridae)—separate diagnostic pathway**	
Pan-filovirus RT-PCR (e.g., L-gene; Altona RealStar Filovirus Screen, CE-IVD)	Broadly reactive; detected the divergent BDBV variant (8/13 INRB samples, May 2026); recommended for BDBV screening [3,21,22]
Zaire-specific RT-qPCR	FALSE NEGATIVE for BDBV (all 29 samples negative, 2007 outbreak); must not be used alone to exclude BDBV [10]
Ebolavirus species-differentiating assays	Required to distinguish BDBV from other ebolaviruses [10,17]
L-gene/NP/VP40-targeted assays	Targets for species-level detection and confirmation [17]
Antigen-capture ELISA (broadly reactive)	Used in the 2007 BDBV discovery when species-specific PCR failed [10]
Sequencing confirmation	Confirms species given >30% glycoprotein divergence from Zaire ebolavirus [17]

**Table 5:** Therapeutic pipeline for ANDV and BDBV.

Drug/Approach	ANDV Status	BDBV Status
Favipiravir	MOST ADVANCED: In vitro activity, animal models, human safety data from COVID-19. Considered for post-exposure prophylaxis and early disease [2]	Not specifically studied; may be relevant if used clinically
Ribavirin	NOT PRIORITIZED: Modest efficacy (CFR ~38–40% vs. ~50% untreated); inconsistent data and safety concerns [23]	Not recommended for current outbreak
Tocilizumab (IL-6 antagonist)	COMPASSIONATE USE in progress: Targets IL-6-driven hyperinflammation in severe disease [16]	Not specifically evaluated; may be relevant if available
Icatibant (bradykinin antagonist)	INVESTIGATIONAL: Targets bradykinin-mediated vascular leak identified in 33-patient cytokine profiling [2]	Potentially relevant if bradykinin pathway involved, but not yet studied
Other antivirals (molnupiravir, baloxavir)	REQUIRE URGENT in vitro evaluation [2]	Not specifically evaluated

**Table 6:** Vaccine development status for ANDV and BDBV.

Vaccine Platform/Status	ANDV	BDBV
DNA vaccine (USAMRIID)	PHASE 1 COMPLETED: multi-dose, needle-free injection, targets ANDV glycoprotein; antibody-mediated protection in hamster models; safety & immunogenicity data published [24]	Preclinical only
Viral vector-based vaccine	PRECLINICAL: In development [2]	PRECLINICAL: Platforms exist (e.g., MVA, Ad26) but BDBV-specific development not yet initiated [3]
mRNA vaccine	PRECLINICAL: In early-stage development [2]	PRECLINICAL: Platform available but BDBV-specific development needed
Cross-protection from existing Ebola vaccines	Not applicable	UNCERTAIN, LIKELY LIMITED: ERVEBO and Zabdeno/Mvabea are Zaire-based; 35% BDBV GP divergence may reduce cross-neutralization; BDBV-specific immunogenicity/protection data required [3]

## Data Availability

No new data were created or analyzed in this study. Data sharing is not applicable to this article.

## Abbreviations

The following abbreviations are used in this manuscript:

ANDV	Andes virus
BDBV	Bundibugyo virus
BVD	Bundibugyo virus disease
CFR	Case fatality rate
ECMO	Extracorporeal membrane oxygenation
GP	Glycoprotein
HFRS	Haemorrhagic fever with renal syndrome
HPS	Hantavirus pulmonary syndrome
IL-6	Interleukin-6
IRF-3	Interferon regulatory factor 3
NP	Nucleoprotein
PHEIC	Public Health Emergency of International Concern
R0	Basic reproduction number
RT-qPCR	Reverse transcription quantitative polymerase chain reaction
SAR	Secondary attack rate
SNV	Sin Nombre virus
USAMRIID	United States Army Medical Research Institute of Infectious Diseases
VLP	Virus-like particle
WHO	World Health Organization
